# Identification and Differentiation of *Polygonum multiflorum* Radix and *Polygoni multiflori* Radix Preaparata through the Quantitative Analysis of Multicomponents by the Single-Marker Method

**DOI:** 10.1155/2019/7430717

**Published:** 2019-08-08

**Authors:** Ding-Qiang Luo, Pu Jia, Shan-Shan Zhao, Ye Zhao, Hai-Jing Liu, Feng Wei, Shuang-Cheng Ma

**Affiliations:** ^1^Shaanxi Institute for Food and Drug Control, Xi'an 710065, China; ^2^Northwest University, Xi'an 710069, China; ^3^Shaanxi University of Chinese Medicine, Xianyang 712046, China; ^4^National Institute for Food and Drug Control, Beijing 100050, China

## Abstract

The quantitative analysis of multicomponents by the single-marker (QAMS) method was established and the relationship between *F* value (the ratio of the sum of the contents of emodin-8-O-*β*-D-glucopyranoside and physcion-8-O-*β*-D-glucopyranoside to the sum of the contents of emodin and physcion) and the steaming time was found to identify and differentiate *Polygonum multiflorum* Radix and its processed product. Emodin was considered as the control substance, and the correction factors of physcion, emodin-8-O-*β*-D-glucopyranoside, and physcion-8-O-*β*-D-glucopyranoside were computed. In addition, the contents of the four components were determined. When the *F* value is greater than or equal to 1.0, the sample was identified as *Polygonum multiflorum* Radix, and if the *F* value was between 0.6 and 1.0, the sample of *Polygoni multiflori* Radix Preaparata was processed incompletely. The *F* value of the qualified Radix *Polygonum multiflorum* should be no more than 0.6. However, the influence of different sample injection volumes and the chromatographic columns and instruments used on the durability of the correction factors and RSD ≤3% hindered accurate identification; therefore, a QAMS method using an external standard value with methodological verification was developed. We redefined the “*Polygonum multiflorum* rules.” The method using “*Polygonum multiflorum* rules” revised after optimization of the determination results was used, as it was accurate and led to convenient operation and low inspection costs, and moreover, the method could differentiate *Polygoni multiflori* Radix Preaparata and *Polygonum multiflorum* Radix medicinal samples and precisely identify samples that were different from the completely processed product *Polygoni multiflori* Radix Preaparata.

## 1. Introduction

The processed product of *Polygonum multiflorum* Radix, *Polygoni multiflori* Radix Preaparata, has several beneficial effects: it nourishes the liver and kidneys, improves blood parameters, strengthens bones and muscles, resolves dampness, and reduces fat content. *Polygonum multiflorum* Radix is known to have detoxifying, carbuncle elimination, malaria prevention, and bowel relaxation effects [1]. The clinical applications of *Polygonum multiflorum* Radix were first recorded in the *Book of Polygonum multiflorum* written by Li during the reign of the Tang dynasty. *Polygonum multiflorum* Radix is sweet and warm and has no toxic effects; it is used to treat anal hemorrhoids and invertebrate diseases of the waist and abdomen, as well as to eliminate colds and promote rib growth. It boosts essence and fertility, enhances appetite, improves physical strength, and revives the skin to delay senescence [2]. *Polygonum multiflorum* Radix has completely different efficacy from that of *Polygoni multiflori* Radix Preaparata. *Polygoni multiflori* Radix Preaparata has been clinically used in large quantities as a bulk traditional Chinese medicine, and previous studies have reported on its serious adverse effects such as hepatotoxicity [3–6]. Some studies have shown that the hepatotoxicity caused by *Polygonum multiflorum* may be related to the toxic components present in it, the processing technology used for extraction, and defects in hepatic enzymes or genes in patients [7–10]. Among 42 studies on these herbs, 19 do not mention the toxicity of *Polygonum multiflorum*, 20 indicate that it is nontoxic, and 3 have recorded its toxicity. Ancient books have described the plantation, harvest, processing, compatibility, and prohibition of consumption of *Polygonum multiflorum* Radix in detail, and most of the previous studies suggest that the processing method used may affect the efficacy or safety of *Polygonum multiflorum* Radix [11]. “Steaming and drying in the sun nine times” (The “nine times” means “long time”) is used to process traditional Chinese medicines such as *Polygonum multiflorum* Radix, *Rehmannia*, and *Polygonatum sibiricum*.

At present, some Chinese medicinal enterprises have simplified *Polygonum multiflorum* Radix processing technologies to reduce the cost, and modern pharmacological experiments have found that the damaging effects of *Polygoni multiflori* Radix Preaparata on the mouse liver are significantly lower than those caused by raw *Polygonum multiflorum* Radix; this indicates that processing can effectively reduce the hepatotoxicity of *Polygonum multiflorum* Radix [12–16]. Experiments showed that the crude sample had higher hepatoxicity than *Polygoni multiflori* Radix Preaparata which was processed by the same batch of *Polygonum multiflorum* Radix according to the process specifications. However, there were 14 batches of *Polygoni multiflori* Radix Preaparata samples which bought from the market had the same or even higher injury effect than the control of *Polygonum multiflorum* Radix, reflecting that the unqualified *Polygoni multiflori* Radix Preaparata which were not processed completely can not effectively reduce the hepatotoxicity of *Polygonum multiflorum* Radix [12]. Information from the Chinese National Knowledge Infrastructure database has reported 686 adverse events of hepatoxicity in the last five years. Therefore, it is necessary to establish a modern method to determine whether *Polygoni multiflori* Radix Preaparata is completely processed to eliminate toxic effects.

Previous studies have analyzed the quality of *Polygonum multiflorum* Radix based on the relationship among 2,3,5,4′-tetrahydroxy diphenyl ethylene-2-O-*β*- glucoside, combined anthraquinones, and calcium oxalate [17] by determining the contents of glucosides and combined anthraquinones in *Polygonum multiflorum* Radix using HPLC-DAD [18]; determining the contents of 2,3,5,4′- tetrahydroxy diphenyl ethylene-2-O-*β*- glucoside, emodin, and emodin monemethyl ether in *Polygonum multiflorum* Radix and its residues using HPLC [19]; determining the contents of five anthraquinones, aloe-emodine, rheine, emodine, chrysophanol, and physcione in *Polygonum multiflorum* using HPLC [20]; determining the contents of four phenols, gallic acid, trans-2,3,5,4′-tetrahydroxystilbene-2-O-*β*-D-glucopyranoside, emodin, and emodin-8-O-*β*-D-glucopyranoside in *Polygonum multiflorum* and its processed form using UHPLC-MS/MS [21]; determining the contents of 2,3,5,4′-tetrahydroxystilbene-2-O-*β*-D-glucoside, emodin-8-O-*β*-D-glucoside, emodine, and physcion using LC-VWD-MS, UPLC-PDA, and HPLC-PAD [22–24] comparing the compositions of *Polygonum multiflorum* Thunb. and *Polygonum multiflorum* Radix using UHPLC-Q-TOF-MS [25]; and determining the 14 main components of *Polygonum multiflorum* Radix collected from different areas as well as the amounts of stibene glucosides, phenolic acids, flavones, and anthraquinones present in *Polygonum multiflorum* Radix using LC-MS/MS [26, 27]. One study showed that after processing of *Polygonum multiflorum* Radix, the contents of two anthraquinones, emodin-8-O-*β*-D-glucopyranoside and emodin monemethyl ether-8-O-*β*-D-glucopyranoside, were decreased in *Polygoni multiflori* Radix Preaparata, but the contents of emodin and emodin monemethyl ether increased [28–30].

However, these methods of analysis could not establish an index to differentiate *Polygonum multiflorum* Radix from *Polygoni multiflori* Radix Preaparata based on compositional variation owing to many differences in the medicinal material obtained from different production areas. The compositional content is involved by the geographical environment; the *Polygonum multiflorum* Radix samples may also meet the content standard of *Polygoni multiflori* Radix Preaparata sometimes. It is irrational to evaluate the quality of *Polygoni multiflori* Radix Preaparata through absolute values. In this study, we steamed 6 batches of *Polygonum multiflorum* Radix for different time periods to observe the variation of *F* values and to find the *F* values when the samples were processed completely and then the samples of *Polygoni multiflori* Radix Preaparata from the market were evaluated through *F* values to judge whether they had been processed completely.

The authors of the present study undertook a special quality analysis project of the Chinese herbal medicine *Polygoni multiflori* Radix Preaparata (*Polygonum multiflorum* Radix) in the Food and Drug Administration in 2015 and put forward “*Polygonum multiflorum* rules” for the first time in the report titled “Quality Analysis of *Polygonum multiflorum* Radix” in 2016 [31], which was used to differentiate *Polygoni multiflori* Radix Preaparata from *Polygonum multiflorum* Radix. However, as four control components, i.e., edomin-8-O-*β*-D-glucopyranoside, physcion-8-O-*β*-D-glucopyranoside, emodin, and physcion, were used, the identification method incurred high costs and had complicated operations. Furthermore, “*Polygonum multiflorum* rules” cannot judge whether the *Polygoni multiflori* Radix Preaparata samples had been processed completely. Therefore, the QAMS method was used to compute the contents of four anthraquinone components, and emodin was considered as the control. Importantly, this study established the method to differentiate *Polygoni multiflori* Radix Preaparata which had been processed completely and thus perfected the “*Polygonum multiflorum* rules.”

In this study, we aimed to differentiate *Polygonum multiflorum* Radix from *Polygoni multiflori* Radix Preaparata using the QAMS method with emodin as the control substance and correction factors for physcion, emodin-8-O-*β*-D-glucopyranoside, and physcion-8-O-*β*-D-glucopyranoside.

## 2. Materials and Methods

### 2.1. Chemicals and Reagents

Both the emodin control sample (110756-201512, purity 98.7%) and the physcion control sample (110758-201415, purity 99.1%) were provided by the National Institute for Food and Drug Control; the emodin-8-O-*β*-D-glucopyranoside control sample (purity 97.0%) and the physcion-8-O-*β*-D-glucopyranoside control sample (purity 98.0%) were purchased from Shanghai Tauto Biotech Co., Ltd. Acetonitrile and methanol were of chromatographical grade, and the water used was ultrapure. All other reagents used were of analytical grade.

### 2.2. Samples

*Polygoni multiflori* Radix Preaparata and *Polygonum multiflorum* Radix samples were spot checked according to the national special spot check plan using the “medicinal material and medicinal piece sampling method” in Appendix 0211 of the fourth version of the *Chinese Pharmacopoeia*. Excluding samples from Hong Kong, Macao, and Taiwan, 172 batches of *Polygonum multiflorum* Radix and *Polygoni multiflori* Radix Preaparata medicinal samples from 22 provinces, 5 autonomous regions, 4 municipalities directly under the central government, and 31 provincial-level administration regions were put under spot check, where there were 66 batches of *Polygonum multiflorum* Radix medicinal samples, involving 51 production enterprises distributed in 19 provinces, and 106 batches of *Polygoni multiflori* Radix Preaparata medicinal samples, involving 100 production enterprises distributed in 26 provinces. The 35 batches of samples in different steaming time periods were obtained from Shaanxi Guangjitang Pharmaceutical Group Co., Ltd.

### 2.3. Instrumentation and Chromatographic Conditions

The following instruments were used: BS224S electronic analytical balance (Sartorius, Germany) and BP211D electronic analytical balance (Sartorius, Germany); an AE 240 electronic analytical balance (Mettler Toledo, Switzerland); Shimadzu LC 2030 3D HPLC (Shimadzu, Japan); Empower chromatographic working station; and Waters e2695 HPLC (Waters, US).

**Figure 1:**
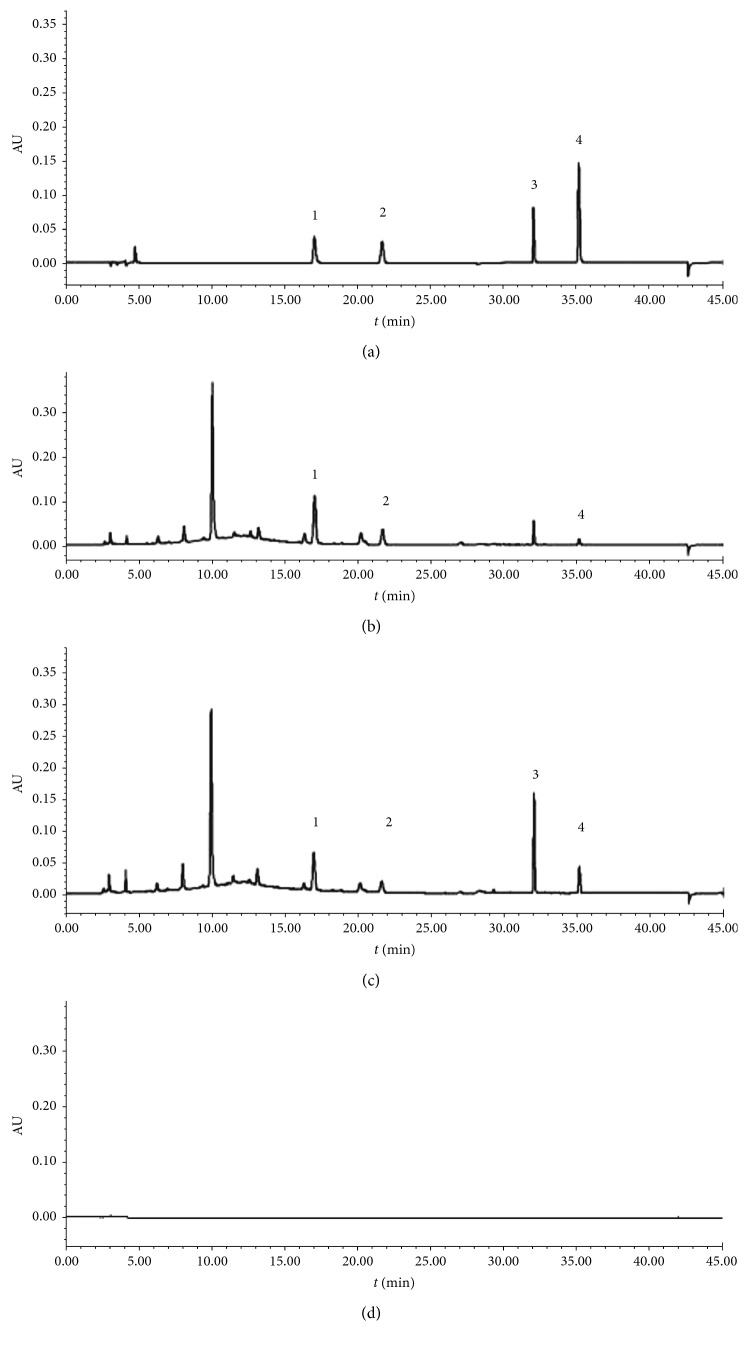
HPLC chromatograms. (a) Mixed control sample. (b) *Polygonum multiflorum* Radix. (c) *Polygoni multiflori* Radix Preaparata. (d) Blank methanol. 1, emodin-8-O-*β*-D-glucopyranoside; 2, physcion-8-O-*β*-D-glucopyranoside; 3, emodin; 4, physcion.

Phenomenex C_18_ bonded silica gel (Phenomenex C_18_, 250 mm × 4.6 mm, 5 *μ*m) was used as the filling agent. The mobile phases included phase A, acetonitrile, and phase B, 0.1% phosphoric acid solution, under gradient elution. The change in the proportion of the mobile phases is shown in Table S1. The other chromatographic conditions were as follows: detection wavelength: 254 nm; flow velocity: 1.0 mL·min; column temperature: 35°C; and sample injection volume: 10 *μ*L. The number of theoretical plates was at least 3,000 for emodin computation. The control samples and their HPLC chromatograms are presented in Figure 1.

### 2.4. Preparation of the Control Sample Solutions

In total, 10.08 mg of the physcion control sample was precisely weighed and placed in a 100-mL measuring flask. The sample was dissolved by adding methanol, with the volume maintained constant to the scale, and was then subjected to shaking and termed “No. 1 stock solution.” Next, 7.20 mg of emodin-8-O-*β*-D-glucopyranoside and 9.92 mg of emodin monemethyl ether-8-O-*β*-D- glucopyranoside were placed in a 100-mL measuring flask, and then, the sample was dissolved by adding methanol; the volume was maintained constant to the scale, and then was subjected to shaking and termed “No. 2 stock solution.” Subsequently, 1 mL of the No. 1 stock solution and 1 mL of the No. 2 stock solution were precisely weighed, placed in a 50-mL measuring flask, diluted by using methanol up to the scale, and shaken to obtain No. 1 control sample solution. Then, 2 mL of the No. 1 stock solution and 2 mL of the No. 2 stock solution were precisely weighed, placed in a 50-mL measuring flask, and diluted by using methanol to the scale to obtain the No. 2 control sample solution. Furthermore, 2 mL of the No. 1 stock solution and 2 mL of the No. 2 stock solution were precisely weighed and placed in a 25-mL measuring flask, and the volume was maintained constant by using methanol and termed “No. 3 control sample solution.” Then, 1 mL of the No. 1 stock solution and 1 mL of the No. 2 stock solution were placed in a 10-mL measuring flask, and the mixture was diluted to the scale to obtain No. 4 control sample solution. Additionally, 2 mL of the No. 1 stock solution and 2 mL of the No. 2 stock solution were placed in a 10-ml measuring flask, and the mixture was diluted to the scale to obtain No. 5 control sample solution; 2 mL of the No. 1 stock solution and 2 mL of the No. 2 stock solution were placed in a 5-mL measuring flask, and the mixture was diluted to the scale to obtain the No. 6 control sample solution. Next, 5 mL of the No. 1 stock solution and 5 mL of the No. 2 stock solution were placed in a 100 mL measuring flask, and the mixture was diluted to the scale to obtain the No. 7 control sample solution.

### 2.5. Preparation of Sample Solutions to Be Tested

An appropriate amount of the samples to be tested was crushed and passed through a No. 4 screen (250 *µ*m ± 9.9 *µ*m). Next, 1 g of the sample powder was precisely weighed and placed in a conical flask with accurate addition of 50 mL of methanol, subjected to heating reflux for 1 h, and then cooled. Methanol was used to supplement for weight loss, and the filtrate obtained was considered as the sample for testing.

### 2.6. Method Validation

#### 2.6.1. Linearity Range

The prepared control sample solutions, 1, 2, 3, 4, 5, 6, and 7 (10 *μ*L each), were used for liquid chromatography, based on the previously mentioned chromatographic conditions. The areas of the chromatographic peaks were recorded; the injection volume (*X*, *μ*g) was considered as the *X*-coordinate, and the peak area (*Y*) was considered as the *Y*-coordinate to obtain standard curves.

#### 2.6.2. Precision Test

The No. 6 mixed control sample solution (10 *μ*L) was precisely absorbed, and sample injection was implemented 6 times.

#### 2.6.3. Stability Testing

The same batch of *Polygonum multiflorum* Radix sample solutions were taken and subjected to chromatography at 0, 2, 4, 6, 12, 18, and 24 h.

#### 2.6.4. Repeatability Test

A sample of the same batch of *Polygonum multiflorum* Radix sample powder (1 g) was taken and divided into 6 parallel portions. Sample solutions were prepared based on the laws and average mass fractions of emodin, physcion, emodin-8-O-*β*-D-glucopyranoside, and physcion-8-O-*β*-D-glucopyranoside, which were 4.256, 1.502, 2.008, and 0.679 mg·g^−1^, respectively.

#### 2.6.5. Recovery Test

Next, 0.5 g of the same batch of *Polygonum multiflorum* Radix medicinal samples was taken, and 2 mL was precisely added into the mixed control sample solution (emodin 1.362 mg·mL^−1^, physcion 0.5595 mg·mL^−1^, emodin-8-O-*β*-D-glucopyranoside 0.64 mg·mL^−1^, and physcion-8-O-*β*-D-glucopyranoside 0.163 mg·mL^−1^). The mixture was then divided into 6 parallel portions, and the solvent was evaporated to dryness. Preparation and determination were conducted according to the same method for samples to be tested, and the recoveries were computed.

### 2.7. Computation of Relative Correction Factors and Relative Retention Time (RRT)

In total, 10 *μ*L of the No. 6 control sample solution was subjected to chromatography, with sample injection 3 times, and the areas of the chromatographic peaks and retention time were recorded. Emodin was considered as the internal standard, and the correction factors for physcion, emodin-8-O-*β*-D-glucopyranoside, and physcion-8-O-*β*-D-glucopyranoside were computed according to formula (1), with their values of 1.09, 0.44, and 0.49, respectively. *A*_*k*_ is the peak area of the internal standard substance, *W*_*k*_ is the mass or concentration of the internal standard substance, *A*_*m*_ is the peak area of the other components *m*, and *W*_*m*_ is the mass or concentration of the other components *m*:

(1) $$\begin{array}{c} f_{km}=\frac{f_{k}}{f_{m}}=\left(\frac{W_{k}\times A_{m}}{W_{m}\times A_{k}}\right). \end{array}$$

The retention time of emodin was considered as 1.00; the RRTs of physcion, emodin-8-O-*β*-D-glucopyranoside, and physcion-8-O-*β*-D-glucopyranoside were computed as 1.06, 0.47, and 0.60, respectively.

### 2.8. Investigation of the Durability of Correction Factors

The influence of different sample injection volumes, different chromatographic columns, and different instruments used for correction factors was investigated.

### 2.9. Determination Method

The sample to be tested and the control sample solution (10 *μ*L each) were subjected to liquid chromatography, and the chromatograms were recorded. The peak area of the emodin control sample and contents of emodin monomethyl ether, emodin-8-O-*β*-D-glucopyranoside, and physcion-8-O-*β*-D-glucopyranoside were computed according to corresponding correction factors.

### 2.10. Comparison of the Determination Results Using QAMS and the External Standard Method

Ten batches of the samples were taken and evaluated using the sample solutions prepared. The external standard method and the QAMS method were used to compute contents and *F* values of emodin, physcion, emodin-8-O-*β*-D-glucopyranoside, and physcion-8-O-*β*-D- glucopyranoside in the test samples.

## 3. Results

### 3.1. Method Validation

Four standard curves showed favorable linearity within the linearity range (Table S2).

The limits of detection of emodin, physcion, emodin-8-O-*β*-D-glucopyranoside, and physcion-8-O-*β*-D-glucopyranoside were 0.00234 *μ*g, 0.0041 *μ*g, 0.00422 *μ*g, and 0.00404 *μ*g, respectively. The limits of quantification of emodin, physcion, emodin-8-O-*β*-D-glucopyranoside, and physcion-8-O-*β*-D-glucopyranoside were 0.00585 *μ*g, 0.0102 *μ*g, 0.0106 *μ*g, and 0.0101 *μ*g, respectively.

The areas of the chromatographic peaks were recorded, and RSD <1% indicated good instrument precision (Table S3). And RSDs of the intraprecision and interprecision for four different components were all lower than 1%. These results showed that the development method has excellent precision.

At 0, 2, 4, 6, 12, 18, and 24 h, the peak areas were recorded, and RSD <2% indicated that the sample solution was stable under room temperature within 24 h (Table S4).

Sample solutions were prepared based on the laws and average mass fractions of emodin, physcion, emodin-8-O-*β*-D-glucopyranoside, and physcion-8-O-*β*-D-glucopyranoside, which were 4.256, 1.502, 2.008, and 0.679 mg·g^−1^, respectively; the RSDs were 2.38%, 2.51%, 1.69%, and 2.28%, respectively, with favorable repeatability (Table S5).

The recovery rates of emodin, physcion, emodin-8-O-*β*-D-glucopyranoside, and physcion-8-O-*β*-D-glucopyranoside were 98.94%, 98.53%, 103.64%, and 106.80%, respectively, and the RSDs were 1.57%, 5.73%, 1.78%, and 3.21%, respectively, indicating accurate results (Tables S6∼S9).

### 3.2. Investigation of the Durability of Correction Factors

The influence of different sample injection volumes, different chromatographic columns, and different instruments used for correction factors was investigated, and all RSDs were found to be less than 3% (Tables 1 and 2).

### 3.3. Results of RRTs

The RRTs and the relative correction factors are presented in Table 3, and their RRTs were within ±5% range of the stipulated value (if the deviation in the RRT exceeded 5%, then the corresponding control sample was substituted).

### 3.4. Comparison of the Determination Results Using QAMS and the External Standard Method

The *F* value (*Polygonum multiflorum* rules) was obtained via the amount of emodin-8-O-*β*-D-glucopyranoside and physcion-8-O-*β*-D-glucopyranoside vs the amount of emodin and physcion. The external standard method and the QAMS method were used to compute contents and *F* values of emodin, physcion, emodin-8-O-*β*-D-glucopyranoside, and physcion-8-O-*β*-D- glucopyranoside in the test samples (Table 4).

### 3.5. Relationship between *F* Value and Steaming Time

#### 3.5.1. Using the QAMS Method to Compute *F* Values

**Figure 2:**
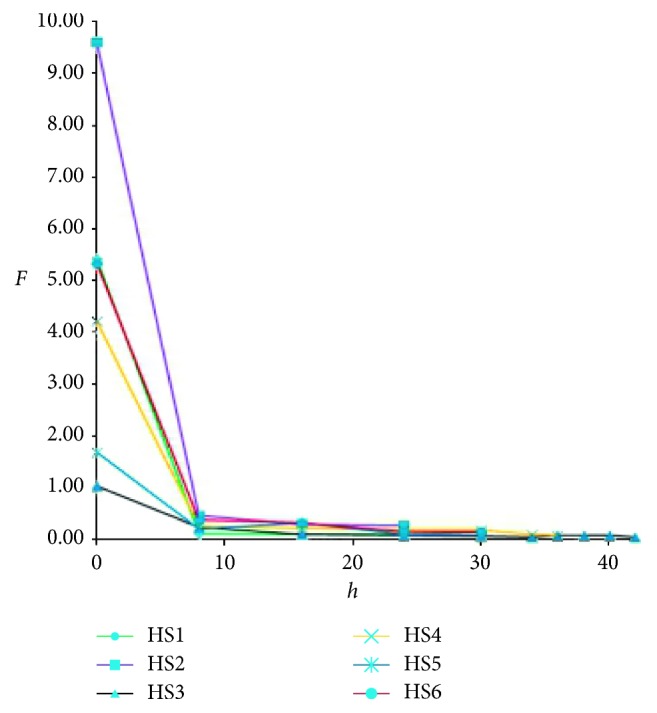
Curve of *F* value and steaming time for *Polygonum multiflorum* Radix and *Polygoni multiflori* Radix Preaparata.

Six batches of *Polygonum multiflorum* Radix samples were produced, and the QAMS method was used to compute *F* values in different steaming time periods (Table 5, Figure 2).

The *F* value is in direct relationship to the steaming time. The *F* values of 6 batches of *Polygonum multiflorum* Radix samples are between 1.0∼10.0; they all decreased to 0.6 and remained stable when we steamed the 6 batches of samples for 8 hours according to the process specifications. Combining the results of our research, the *F* values of the qualified *Polygoni multiflori* Radix Preaparata samples are no more than 0.6. When the value is more than 0.6, it is suggested that the steaming time is not enough, and the samples could have the property of the crude.

## 4. Application

Using the QAMS method, *F* values of 66 batches of *Polygonum multiflorum* Radix samples and 106 batches of *Polygoni multiflori* Radix Preaparata samples (Table 6) were computed and the quality of the *Polygoni multiflori* Radix Preaparata from the market (Table 7) was evaluated. The *F* values of *Polygonum multiflorum* Radix samples were observed to judge whether the limitation (*F* ≥ 1.0) of the crude is reasonable.

The established method (the *F* value of qualified *Polygoni multiflori* Radix Preaparata is no more than 0.6.) is used to analyze *Polygoni multiflori* Radix Preaparata samples of the market, and the qualified rate is 89.4%. The values of *Polygonum multiflorum* Radix are all greater than or equal to 1.0 which shows that the limitation of *Polygonum multiflorum* Radix (*F* ≥ 1.0) is reasonable.

## 5. Discussion

In this study, the ratio of the sum of the contents of emodin-8-O-*β*-D-glucopyranoside and physcion-8-O-*β*-D-glucopyranoside to the sum of the contents of emodin and physcion (*F* values) was computed to differentiate *Polygonum multiflorum* Radix medicinal samples from *Polygoni multiflori* Radix Preaparata medicinal samples. The *F* values of *Polygonum multiflorum* Radix medicinal samples should be greater or equal to 1.0, and those of *Polygoni multiflori* Radix Preaparata medicinal samples should be no more than 0.6.

As a medicine that can prolong life, *Polygoni multiflori* Radix Preaparata has been widely used in TCM clinics, and reports on its hepatotoxicity have increased in recent years. *Polygoni multiflori* Radix Preaparata-processed products are produced using “steaming and drying in the sun for nine times,” a traditional processing method. This process is complicated, time- and energy-consuming, toxicity-reducing, and efficacy-enhancing, and the properties of raw *Polygonum multiflorum* Radix are altered. Pharmacological experiments also indicate that *Polygoni multiflori* Radix Preaparata has a significantly lower damaging effect on the mouse liver than raw *Polygonum multiflorum* Radix. At present, some medicine production enterprises have simplified processing technologies and reduced production cost by violating laws and regulations; consequently, incompletely processed *Polygoni multiflori* Radix Preaparata samples have flooded the market. It is not easy to discriminate products containing incompletely processed *Polygoni multiflori* Radix Preaparata; therefore, it is necessary to establish a key method to differentiate *Polygonum multiflorum* Radix from *Polygoni multiflori* Radix Preaparata so as to prevent inferior *Polygoni multiflori* Radix Preaparata samples from entering the market.

Determining whether *Polygoni multiflori* Radix Preaparata has been completely processed has always been difficult within the industry. In a special project of the National Institute for Food and Drug Control, *Polygoni multiflori* Radix Preaparata (*Polygonum multiflorum* Radix) Quality Analysis, 172 batches of samples were collected and their quality was evaluated using a standard test and exploratory analysis. Mass data analysis and documented studies in 2016 [22–24] showed that as processing times of raw *Polygonum multiflorum* Radix increased, the contents of anthraquinones decreased, whereas those of anthraquinone aglycones increased. Four components, i.e., emodin, emodin-8-O-*β*-D-glucopyranoside, physcion, and physcion-8-O-*β*-D-glucopyranoside, were evaluated in this study, and the total content of emodin-8-O-*β*-D-glucopyranoside and physcion-8-O-*β*-D-glucopyranoside was higher than the total content of emodin and physcion in *Polygonum multiflorum* Radix samples of the same batch. The total content of emodin-8-O-*β*-D-glucopyranoside and physcion-8-O-*β*-D-glucopyranoside was lower than the total content of emodin and physcion in completely processed *Polygonum multiflorum* Radix samples. As the content of the four components greatly differed between *Polygonum multiflorum* Radix and *Polygoni multiflori* Radix Preaparata samples obtained from different production areas, it was difficult to use a concrete content limit to differentiate *Polygonum multiflorum* Radix from *Polygoni multiflori* Radix Preaparata, which is a drawback and has remained unsolved previously. Through a mass data analysis in 2016, the “*Polygonum multiflorum* rules” theory was proposed for the first time in a thesis [25], and the *F* value was used to differentiate *Polygonum multiflorum* Radix from *Polygoni multiflori* Radix Preaparata. As four control samples, emodin, physcion, emodin-8-O-*β*-D-glucopyranoside, and physcion-8-O-*β*-D-glucopyranoside, were used in this method, it had disadvantages such as high cost and complicated operations; in addition, the limit value was set as 1, which caused misjudgment. Furthermore, “*Polygonum multiflorum* rules” cannot judge whether the *Polygoni multiflori* Radix Preaparata samples had been processed completely.

To overcome this problem, low-cost emodin was used as the control sample in the “QAMS” method, which was designed to compute correction factors of emodin-8-O-*β*-D-glucopyranoside, physcion-8-O-*β*-D-glucopyranoside, and physcion, and the contents of four anthraquinone components in *Polygoni multiflori* Radix Preaparata and *Polygonum multiflorum* Radix were computed. This is the first time that the QAMS method has been used to differentiate *Polygonum multiflorum* Radix and *Polygoni multiflori* Radix Preaparata. To investigate the durability of the correction factors, different sample injection volumes within 2–30 *µ*L were adopted, and the RSD of the correction factor for each component was set as ≤2%; HPLC and C_18_ chromatographic columns were used to investigate the robustness of the correction factors, and the results showed that the RSD of each component was not greater than 3%. A comparison of results using the QAMS method and those using an external standard method from 10 batch samples showed no significant difference. Methodological validation established that the QAMS method is feasible and can effectively solve problems of previously used identification methods such as the high costs and multiple operative steps involved; additionally, the limited *F* value of the “*Polygonum multiflorum* rules” was revised that *F* value of the qualified *Polygoni multiflori* Radix Preaparata should be no more than 0.6, which effectively solved misjudgment after significant figure revision. Importantly, we established the method to differentiate *Polygoni multiflori* Radix Preaparata which had been processed completely and thus perfected the “*Polygonum multiflorum* rules.”

## 6. Conclusions

In this study, an emodin control sample was used for the first time to differentiate *Polygonum multiflorum* Radix from *Polygoni multiflori* Radix Preaparata using a QAMS method. In addition to advantages such as low cost and simple and convenient operation, this method could accurately differentiate *Polygonum multiflorum* Radix from *Polygoni multiflori* Radix Preaparata and identify incompletely processed *Polygonum multiflorum* Radix samples so as to prevent substandard products from flooding the market. This method of differentiation ensures the safe and effective use of the medicinal material *Polygonum multiflorum* Radix.

## Figures and Tables

**Table 1:** Correction factors under different sample injection volumes.

Sample injection volume (*μ*L)	Physcion	Physcion-8-O-*β*-D-glucopyranoside	Emodin-8-O*-β*-D-glucopyranoside
2	1.134	0.469	0.428
5	1.127	0.487	0.441
10	1.104	0.486	0.435
20	1.095	0.490	0.438
30	1.096	0.492	0.435
Average value	1.11	0.49	0.44
RSD (%)	1.64	1.71	1.11

**Table 2:** Correction factors for different instruments and chromatographic columns.

Instrument	Chromatographic column	Emodin monemethyl ether	Physcion-8-O-*β*-D-glucopyranoside	Emodin-8-O-*β*-D-glucopyranoside
SHIMADZU LC2030 3D HPLC	Phenomenex C18	1.09	0.49	0.44
	SHIMADZU C18	1.09	0.49	0.44
Waters e2695 HPLC	Phenomenex C18	1.09	0.47	0.45
	SHIMADZU C18	1.08	0.47	0.42
Average value		1.09	0.48	0.44
RSD (%)		0.75	2.41	2.88

**Table 3:** RRTs and relative correction factors.

Components to be tested (peak)	RRT	Relative correction factor
Emodin	1.00	1.00
Physcion	1.06	1.09
Emodin-8-O-*β*-D-glucopyranoside	0.47	0.44
Physcion-8-O-*β*-D-glucopyranoside	0.60	0.49

**Table 4:** Results of the comparison of 10 batch samples.

No.	Emodin	Physcion (*μ*g·g^−1^)		Emodin-8-O-*β*-D-glucopyranoside (*μ*g·g^−1^)		Physcion-8-O-*β*-D-glucopyranoside (*μ*g·g^−1^)		*F* value	
		QAMS	External standard method	QAMS	External standard method	QAMS	External standard method	QAMS	External standard method
*Polygoni multiflori* Radix Preaparata 01	82.348	25.770	24.189	53.922	54.958	10.929	11.074	0.60	0.62
*Polygoni multiflori* Radix Preaparata 02	205.005	74.247	74.110	42.633	42.941	7.903	7.948	0.18	0.18
*Polygoni multiflori* Radix Preaparata 03	29.817	18.137	18.104	6.834	6.883	3.795	3.816	0.22	0.23
*Polygoni multiflori* Radix Preaparata 04	1.714	0.562	0.558	0.386	0.301	0.088	0.079	0.17	0.17
*Polygoni multiflori* Radix Preaparata 05	3.834	1.210	1.303	2.309	1.930	0.684	0.669	0.59	0.51
*Polygoni multiflori* Radix Preaparata 06 (disqualified)	0.246	0.151	0.150	0.566	0.452	0.366	0.363	2.35	2.06
*Polygonum multiflorum* Radix 07	12.148	5.687	5.677	41.728	42.029	35.911	36.112	4.35	4.38
*Polygonum multiflorum* Radix 08	89.144	36.892	36.824	103.343	104.090	48.844	49.119	1.21	1.22
*Polygonum multiflorum* Radix 09	20.369	11.598	11.577	40.942	41.238	32.344	32.526	2.29	2.32
*Polygonum multiflorum* Radix 10	0.174	0.097	0.096	0.604	0.482	0.370	0.333	3.59	3.02

**Table 5:** *F* values of 6 batches of *Polygonum multiflorum* Radix samples in different steaming time periods.

Lot.	Emodin-8-O-*β*-D-glucopyranoside	Emodin monomethyl ether-8-O-*β*-D-glucopyranoside	Emodin	Emodin monomethyl ether	*F*
HS1 (0 h)	0.217	0.053	0.039	0.011	5.41
HS1 (8 h)	0.029	0.010	0.265	0.081	0.11
HS1 (16 h)	0.025	0.009	0.304	0.081	0.09
HS1 (24 h)	0.017	0.007	0.187	0.060	0.10
HS2 (0 h)	0.319	0.109	0.034	0.010	9.61
HS2 (8 h)	0.113	0.040	0.253	0.085	0.45
HS2 (16 h)	0.081	0.029	0.283	0.096	0.29
HS2 (24 h)	0.074	0.029	0.290	0.102	0.26
HS3 (0 h)	0.069	0.026	0.069	0.023	1.03
HS3 (8 h)	0.024	0.008	0.105	0.036	0.23
HS3 (16 h)	0.010	0.005	0.112	0.041	0.10
HS3 (24 h)	0.011	0.005	0.173	0.062	0.07
HS3 (30 h)	0.007	0.003	0.140	0.050	0.05
HS3 (34 h)	0.007	0.005	0.202	0.076	0.04
HS3 (36 h)	0.011	0.006	0.178	0.063	0.07
HS3 (38 h)	0.011	0.005	0.177	0.062	0.07
HS3 (40 h)	0.011	0.007	0.165	0.062	0.08
HS3 (42 h)	0.007	0.005	0.170	0.065	0.05
HS4 (0 h)	0.253	0.089	0.062	0.019	4.21
HS4 (8 h)	0.047	0.015	0.186	0.066	0.24
HS4 (16 h)	0.059	0.018	0.266	0.085	0.22
HS4 (24 h)	0.042	0.012	0.234	0.072	0.18
HS4 (30 h)	0.045	0.015	0.253	0.083	0.18
HS4 (34 h)	0.030	0.011	0.303	0.109	0.10
HS4 (36 h)	0.021	0.008	0.311	0.105	0.07
HS5 (0 h)	0.181	0.063	0.108	0.037	1.68
HS5 (8 h)	0.048	0.012	0.220	0.073	0.20
HS5 (16 h)	0.052	0.016	0.160	0.056	0.31
HS5 (24 h)	0.027	0.008	0.275	0.086	0.10
HS5 (30 h)	0.025	0.008	0.196	0.071	0.12
HS6 (0 h)	0.245	0.085	0.045	0.016	5.33
HS6 (8 h)	0.086	0.023	0.217	0.074	0.38
HS6 (16 h)	0.080	0.024	0.239	0.087	0.32
HS6 (24 h)	0.041	0.011	0.266	0.086	0.15
HS6 (30 h)	0.029	0.008	0.186	0.066	0.15

**Table 6:** *F* values of samples from market.

Name	No.	Emodin-8-O-*β*-D-glucopyranoside	Physcion-8-O-*β*-D- glucopyranoside	Emodin	Physcion	*F*
*Polygonum multiflorum* Radix	001	0.165	0.047	0.013	0.004	12.72
	002	0.382	0.116	0.034	0.011	11.07
	003	5.255	1.954	0.559	0.17	9.89
	004	0.106	0.061	0.012	0.005	9.6
	005	0.338	0.102	0.038	0.011	8.87
	006	3.593	1.474	0.533	0.17	7.21
	007	1.053	0.766	0.189	0.072	6.97
	008	4.36	2.128	0.737	0.21	6.85
	009	1.768	0.862	0.291	0.099	6.74
	010	0.326	0.1	0.057	0.017	5.73
	011	1.456	0.622	0.286	0.079	5.69
	012	1.301	0.527	0.246	0.079	5.62
	013	2.206	0.953	0.45	0.132	5.43
	014	1.575	0.714	0.31	0.115	5.39
	015	2.153	1.007	0.432	0.158	5.36
	016	2.747	0.994	0.528	0.179	5.29
	017	1.292	0.415	0.25	0.075	5.25
	018	4.585	1.853	0.93	0.317	5.16
	019	2.226	0.927	0.483	0.149	4.99
	020	1.488	0.781	0.334	0.125	4.94
	021	2.335	1.051	0.538	0.195	4.62
	022	2.709	1.324	0.686	0.226	4.42
	023	1.378	1.309	0.439	0.184	4.31
	024	3.601	1.446	0.902	0.286	4.25
	025	2.223	0.859	0.584	0.17	4.09
	026	0.922	0.589	0.247	0.123	4.08
	027	2.15	0.79	0.576	0.246	3.58
	028	2.263	1.002	0.709	0.227	3.49
	029	1.369	0.808	0.469	0.17	3.41
	030	0.068	0.042	0.024	0.009	3.33
	031	1.591	0.57	0.432	0.223	3.3
	032	2.235	0.836	0.694	0.249	3.26
	033	0.188	0.07	0.057	0.024	3.19
	034	1.569	1.168	0.33	0.581	3
	035	1.911	0.715	0.658	0.226	2.97
	036	3.089	1.6	1.204	0.411	2.9
	037	3.694	1.407	1.384	0.476	2.74
	038	0.971	0.462	0.297	0.23	2.72
	039	0.822	0.483	0.333	0.156	2.67
	040	0.733	0.284	0.295	0.101	2.57
	041	0.67	0.563	0.332	0.166	2.48
	042	2.894	1.031	1.325	0.379	2.3
	043	2.446	0.916	1.123	0.398	2.21
	044	1.662	0.58	0.752	0.276	2.18
	045	2.693	1.183	1.421	0.425	2.1
	046	1.777	0.735	1.004	0.349	1.86
	047	0.747	0.467	0.448	0.215	1.83
	048	0.085	0.035	0.043	0.024	1.8
	049	1.822	0.585	1.032	0.365	1.72
	050	0.063	0.042	0.045	0.018	1.68
	051	1.499	0.29	0.818	0.255	1.67
	052	0.099	0.039	0.064	0.02	1.65
	053	0.106	0.04	0.069	0.022	1.61
	054	0.104	0.038	0.071	0.021	1.54
	055	1.237	0.447	0.841	0.29	1.49
	056	0.59	0.194	0.404	0.124	1.49
	057	0.141	0.055	0.089	0.046	1.45
	058	1.879	0.712	1.407	0.443	1.4
	059	1.328	0.55	1.05	0.313	1.38
	060	0.072	0.024	0.054	0.017	1.35
	061	1.245	0.481	1.047	0.308	1.27
	062	0.365	0.33	0.361	0.218	1.2
	063	0.935	0.448	0.771	0.441	1.14
	064	0.5	0.185	0.462	0.138	1.14
	065	0.06	0.021	0.059	0.019	1.06
	066	0.092	0.026	0.091	0.03	0.97

*Polygoni multiflori* Radix Preaparata	001	0.044	0.028	0.008	0.004	5.61
	002	2.278	0.889	1.002	0.326	2.38
	003	0.058	0.029	0.031	0.016	1.83
	004	4.461	1.584	2.528	0.796	1.82
	005	2.42	0.774	1.69	0.521	1.44
	006	0.33	0.098	0.228	0.074	1.41
	007	1.651	0.535	1.083	0.482	1.4
	008	0.204	0.06	0.181	0.056	1.12
	009	1.559	0.589	1.387	0.551	1.11
	010	2.229	0.835	2.173	0.72	1.06
	011	2.278	0.784	2.511	0.75	0.94
	012	0.105	0.04	0.109	0.045	0.94
	013	0.108	0.02	0.12	0.037	0.82
	014	0.077	0.029	0.107	0.038	0.73
	015	0.06	0.03	0.089	0.041	0.69
	016	0.697	0.271	1.056	0.383	0.67
	017	0.776	0.247	1.173	0.436	0.64
	018	0.119	0.036	0.188	0.066	0.61
	019	0.454	0.195	0.749	0.326	0.6
	020	1.252	0.364	2.138	0.657	0.58
	021	0.902	0.207	1.391	0.529	0.58
	022	0.1	0.032	0.173	0.055	0.58
	023	1.234	0.446	2.333	0.704	0.55
	024	0.994	0.345	1.897	0.622	0.53
	025	1.378	0.426	2.585	0.865	0.52
	026	0.557	0.197	1.083	0.373	0.52
	027	0.079	0.028	0.152	0.054	0.52
	028	1.93	0.669	3.834	1.303	0.51
	029	0.911	0.332	1.635	0.824	0.51
	030	0.109	0.022	0.194	0.062	0.51
	031	0.058	0.024	0.112	0.049	0.51
	032	0.68	0.16	1.292	0.434	0.49
	033	0.089	0.03	0.183	0.067	0.48
	034	0.073	0.023	0.151	0.049	0.48
	035	0.566	0.193	1.163	0.456	0.47
	036	0.064	0.024	0.14	0.046	0.47
	037	2.114	0.718	4.527	1.622	0.46
	038	0.52	0.223	1.211	0.433	0.45
	039	0.111	0.031	0.236	0.08	0.45
	040	0.116	0.037	0.251	0.085	0.45
	041	0.047	0.015	0.103	0.042	0.43
	042	0.686	0.288	1.699	0.648	0.41
	043	0.081	0.023	0.188	0.062	0.41
	044	0.023	0.009	0.052	0.025	0.41
	045	0.621	0.212	1.648	0.614	0.37
	046	0.14	0.052	0.389	0.132	0.37
	047	0.091	0.027	0.245	0.081	0.36
	048	0.035	0.011	0.09	0.039	0.36
	049	0.882	0.288	2.576	0.899	0.34
	050	0.841	0.288	2.495	0.994	0.32
	051	0.505	0.157	1.545	0.522	0.32
	052	1.064	0.388	3.429	1.152	0.32
	053	0.49	0.211	1.715	0.652	0.3
	054	0.024	0.013	0.079	0.045	0.3
	055	0.848	0.264	2.932	0.968	0.29
	056	0.042	0.013	0.135	0.052	0.29
	057	0.339	0.08	1.1	0.408	0.28
	058	0.075	0.018	0.249	0.081	0.28
	059	0.675	0.12	2.241	0.724	0.27
	060	0.425	0.135	1.571	0.6	0.26
	061	0.148	0.1	0.663	0.341	0.25
	062	0.027	0.01	0.106	0.042	0.25
	063	0.749	0.209	3.037	1.071	0.23
	064	0.174	0.103	0.842	0.454	0.21
	065	0.479	0.143	2.105	0.819	0.21
	066	0.616	0.147	2.67	0.919	0.21
	067	0.471	0.125	2.041	0.831	0.21
	068	0.537	0.126	2.536	0.828	0.2
	069	0.042	0.014	0.2	0.074	0.2
	070	0.04	0.013	0.208	0.073	0.19
	071	0.033	0.009	0.158	0.061	0.19
	072	0.257	0.143	1.331	0.847	0.18
	073	0.23	0.046	1.156	0.375	0.18
	074	0.608	0.246	3.583	1.232	0.18
	075	0.035	0.013	0.196	0.073	0.18
	076	0.096	0.066	0.594	0.336	0.17
	077	0.408	0.101	2.114	0.811	0.17
	078	0.302	0.079	1.714	0.558	0.17
	079	0.129	0.069	0.735	0.449	0.17
	080	0.196	0.055	1.072	0.431	0.17
	081	0.486	0.071	2.493	0.933	0.16
	082	0.168	0.099	1.179	0.543	0.16
	083	0.37	0.11	2.244	0.882	0.15
	084	0.368	0.11	2.331	0.932	0.15
	085	0.096	0.054	0.724	0.341	0.14
	086	0.817	0.139	5.137	1.697	0.14
	087	0.316	0.066	2.107	0.72	0.14
	88	0.243	0.094	1.802	0.708	0.13
	89	0.362	0.087	2.529	0.89	0.13
	90	0.25	0.08	1.788	0.735	0.13
	91	0.089	0.06	0.781	0.384	0.13
	92	0.15	0.065	1.362	0.547	0.11
	93	0.241	0.085	2.075	0.87	0.11
	94	0.286	0.056	2.316	0.832	0.11
	95	0.019	0.006	0.157	0.065	0.11
	96	0.167	0.039	1.451	0.618	0.1
	97	0.118	0.062	1.035	0.819	0.1
	98	0.253	0.061	2.401	0.841	0.1
	99	0.21	0.106	2.884	0.912	0.08
	100	0.24	0.057	3.181	1.072	0.07
	101	0.206	0	2.211	0.765	0.07
	102	0.299	0.045	4.088	1.435	0.06
	103	0.134	0.064	2.892	1.038	0.05
	104	0.052	0	1.522	0.601	0.02
	105	0.083	0	2.913	0.952	0.02
	106	0.075	0	3.283	1.053	0.02

**Table 7:** Qualified rate of *Polygonum multiflorum* Radix and *Polygoni multiflori* Radix Preaparata.

Sample	Totle samples	*F* ≥ 1.0	1.0 ＞ *F* ＞ 0.6	*F* ≤ 0.6	Qualified rate (%)
*Polygonum multiflorum* Radix	66	66	—	—	100.0
*Polygoni multiflori* Radix Preaparata	106	10	6	90	84.9

## Data Availability

The data used to support the findings of this study are included within the article.

## Supplementary Materials

Supplementary Materials Table S1: Gradient elution of the mobile phases. Table S2: Standard curves of the four components. Table S3: Precision test results. Table S4: Stability test results. Table S5: Repeatability test results. Table S6: Recovery test results for emodin. Table S7: Recovery test results for physcion. Table S8: Recovery test results for emodin-8-O-*β*-D-glucopyranoside. Table S9: Recovery test results for physcion-8-O-*β*-D-glucopyranoside.
