# Supplementary material for: Identification and Differentiation of Polygonum multiflorum Radix and Polygoni multiflori Radix Preaparata through the Quantitative Analysis of Multicomponents by the Single-Marker Method
Source: J Anal Methods Chem. 2019 Aug 8;2019:7430717. doi: 10.1155/2019/7430717 (PMC6702820; doi:10.1155/2019/7430717)
Supplement: Supplementary Materials — Table S1: Gradient elution of the mobile phases. Table S2: Standard curves of the four components. Table S3: Precision test results. Table S4: Stability test results. Table S5: Repeatability test results. Table S6: Recovery test results for emodin. Table S7: Recovery test results for physcion. Table S8: Recovery test results for emodin-8-O-β-D-glucopyranoside. Table S9: Recovery test results for physcion-8-O-β-D-glucopyranoside. [file 7430717.f1.doc]

Supplementary Materials

Table S1: Gradient elution of the mobile phases

| Time (min) | A% (acetonitrile) | B% (0.1% phosphoric solution) |
| --- | --- | --- |
| 0 | 18 | 82 |
| 8 | 30 | 70 |
| 24 | 36 | 64 |
| 28 | 80 | 20 |
| 39 | 95 | 5 |
| 40 | 18 | 82 |
| 45 | 18 | 82 |

Table S2: Standard curves of the four components

| Chemical compound | Standard curve | Concentration range/µg | R |
| --- | --- | --- | --- |
| Emodin monemethyl ether | Y= 37467X + 8372.7 | 0.020～0.504 | 0.9998 |
| Emodin | Y = 35039X - 918.01 | 0.014～0.360 | 0.9999 |
| Emodin-8-*O*-β-D- glucopyranoside | Y = 15520X- 2428.3 | 0.022～0.558 | 0.9999 |
| Emodin monemethyl ether -8-*O*-β-D- glucopyranoside | Y = 17587X - 2660 | 0.020～0.496 | 0.9998 |

Table S3 Precision test results

| No. | Peak area of emodin-8-*O*-β-D- glucopyranoside | Peak area of physcion -8-*O*-β-D- glucopyranoside | Peak area of emodin | Peak area of physcion |
| --- | --- | --- | --- | --- |
| 1 | 705466 | 701536 | 1030750 | 1570987 |
| 2 | 705178 | 699901 | 1030502 | 1570928 |
| 3 | 705126 | 699594 | 1031792 | 1571869 |
| 4 | 705261 | 698942 | 1031558 | 1573020 |
| 5 | 704978 | 699905 | 1032552 | 1574398 |
| 6 | 705369 | 698306 | 1033167 | 1574510 |
| RSD% | 0.10 | 0.10 | 0.16 | 0.02 |

Table S4: Stability test results

| Time (h) | Peak area of emodin-8-*O*-β-D- glucopyranoside | Peak area of physcion -8-*O*-β-D- glucopyranoside | Peak area of emodin | Peak area of physcion |
| --- | --- | --- | --- | --- |
| 0 | 703288 | 699705 | 1027102 | 1564042 |
| 2 | 706302 | 702306 | 1031717 | 1572610 |
| 4 | 705178 | 699901 | 1030502 | 1570928 |
| 6 | 705369 | 698306 | 1033167 | 1574510 |
| 12 | 703588 | 695648 | 1031768 | 1573832 |
| 18 | 686621 | 675317 | 1017263 | 1535314 |
| 24 | 693193 | 680625 | 1020169 | 1556139 |
| RSD% | 1.08 | 1.54 | 0.62 | 0.92 |

Table S5 Repeatability test results

| No. | Emodin/mg·g^-1^ | Emodin monomethy l ether /mg·g^-1^ | Peak area of emodin-8-*O*-β-D- glucopyranoside /mg·g^-1^ | Peak area of emodin monomethy l ether -8-*O*-β-D- glucopyranoside /mg·g^-1^ |
| --- | --- | --- | --- | --- |
| 1 | 4.065 | 1.430 | 1.950 | 0.652 |
| 2 | 4.331 | 1.534 | 2.036 | 0.690 |
| 3 | 4.344 | 1.528 | 2.038 | 0.696 |
| 4 | 4.287 | 1.501 | 2.020 | 0.681 |
| 5 | 4.272 | 1.516 | 2.017 | 0.684 |
| 6 | 4.238 | 1.504 | 1.986 | 0.673 |
| Average value | 4.256 | 1.502 | 2.008 | 0.679 |
| RSD/% | 2.38 | 2.51 | 1.69 | 2.28 |

Table S6: Recovery test results for emodin

|  | Sample injection amount/  g | Sample content/mg | Added amount/mg | Measured total amount/mg | Recovery/% | Average recovery/% | RSD/% |
| --- | --- | --- | --- | --- | --- | --- | --- |
| 1 | 0.5284 | 2.249 | 2.724 | 4.905 | 97.50 | 98.94 | 1.57 |
| 2 | 0.5270 | 2.243 | 2.724 | 5.01 | 101.58 |
| 3 | 0.5026 | 2.139 | 2.724 | 4.815 | 98.24 |
| 4 | 0.5138 | 2.187 | 2.724 | 4.860 | 98.13 |
| 5 | 0.5221 | 2.222 | 2.724 | 4.895 | 98.13 |
| 6 | 0.5213 | 2.219 | 2.724 | 4.945 | 100.07 |

Table S7 Recovery test results for physcion

|  | Sample injection amount/  g | Sample content/mg | Added amount/mg | Measured total amount/mg | Recovery/% | Average recovery/% | RSD/% |
| --- | --- | --- | --- | --- | --- | --- | --- |
| 1 | 0.5284 | 0.794 | 1.119 | 1.920 | 100.63 | 98.53 | 3.17 |
| 2 | 0.5270 | 0.792 | 1.119 | 1.900 | 99.02 |
| 3 | 0.5026 | 0.755 | 1.119 | 1.920 | 104.11 |
| 4 | 0.5138 | 0.772 | 1.119 | 1.845 | 95.89 |
| 5 | 0.5221 | 0.784 | 1.119 | 1.855 | 95.71 |
| 6 | 0.5213 | 0.783 | 1.119 | 1.895 | 99.37 |

Table S8: Recovery test results for emodin-8-*O*-*β*-D- glucopyranoside

|  | Sample injection amount/  G | Sample content/mg | Added amount/mg | Measured total amount/mg | Recovery/% | Average recovery/% | RSD/% |
| --- | --- | --- | --- | --- | --- | --- | --- |
| 1 | 0.5284 | 1.061 | 1.28 | 2.370 | 102.26 | 103.64 | 1.78 |
| 2 | 0.5270 | 1.058 | 1.28 | 2.425 | 106.79 |
| 3 | 0.5026 | 1.009 | 1.28 | 2.325 | 102.81 |
| 4 | 0.5138 | 1.032 | 1.28 | 2.350 | 102.97 |
| 5 | 0.5221 | 1.048 | 1.28 | 2.355 | 102.11 |
| 6 | 0.5213 | 1.047 | 1.28 | 2.390 | 104.92 |

Table S9: Recovery test results for physcion-8-*O*-*β*-D- glucopyranoside

|  | Sample injection amount/  g | Sample content/mg | Added amount/mg | Measured total amount/mg | Recovery/% | Average recovery/% | RSD/% |
| --- | --- | --- | --- | --- | --- | --- | --- |
| 1 | 0.5284 | 0.359 | 0.326 | 0.695 | 103.07 | 106.80 | 3.21 |
| 2 | 0.5270 | 0.358 | 0.326 | 0.715 | 109.51 |
| 3 | 0.5026 | 0.341 | 0.326 | 0.685 | 105.52 |
| 4 | 0.5138 | 0.349 | 0.326 | 0.690 | 104.60 |
| 5 | 0.5221 | 0.355 | 0.326 | 0.700 | 105.83 |
| 6 | 0.5213 | 0.354 | 0.326 | 0.720 | 112.27 |
